# Assessment of Cardiovascular Risk Factors in Young Adults through the Nursing Diagnosis: A Cross-Sectional Study among International University Students

**DOI:** 10.3390/healthcare9010091

**Published:** 2021-01-17

**Authors:** Gonzalo Duarte-Clíments, Tibelle Freitas Mauricio, Juan Gómez-Salgado, Rafaella Pessoa Moreira, Macarena Romero-Martín, María Begoña Sánchez-Gómez

**Affiliations:** 1 University School of Nursing, University of La Laguna, Candelaria NS University Hospital, Canary Islands Health Service, 38010 Santa Cruz de Tenerife, Spain; extgduartcl@ull.edu.es (G.D.-C.); extmsanchez@ull.edu.es (M.B.S.-G.); 2 Instituto Federal de Educação, Ciência e Tecnologia do Ceará, Fortaleza 60410-426, Brazil; tibelle.mauricio@ifce.edu.br; 3 Department of Sociology, Social Work and Public Health, Faculty of Labour Sciences, University of Huelva, 21007 Huelva, Spain; 4 Safety and Health Postgraduate Programme, Universidad Espíritu Santo, Guayaquil 092301, Ecuador; 5 Health Sciences Institute, University for International Integration of the Afro-Brazilian Lusophony (UNILAB), Redenção 62790-000, Ceará, Brazil; rafaellapessoa@unilab.edu.br; 6 Department of Nursing, University of Huelva, 21007 Huelva, Spain; macarena.romero@denf.uhu.es

**Keywords:** cardiovascular function, cardiovascular risk, university students, young adulthood, developmentally appropriate healthcare, transitional care

## Abstract

Four out of five deaths from cardiovascular disease are due to heart attacks and strokes in low- and middle-income countries. Early identification of risk factors in exposed individuals will help to develop interventions that may eliminate and/or reduce these risks and prevent the development of cardiovascular diseases. So, it is necessary to investigate the risk of impaired cardiovascular function in university students due to the increase in some risk factors and cardiovascular events in young adults, and to describe its epidemiology among international university students. For this, an observational cross-sectional study through interviews is designed. The clinical validity was addressed following the Fehring model. In addition, anthropometric data and results of laboratory tests were collected. The nursing diagnosis “Risk of impaired cardiovascular function” showed clinical validity, high sensitivity and specificity, as well as predictive values. Fehring ratio values were above 0.79 and Kappa Index above 0.72. The study showed a high frequency of this nursing diagnosis among university students, especially in students of Brazilian nationality. The main risks of impaired cardiovascular function found in 86.8% of students were: family history of cardiovascular disease, sedentary lifestyle, pharmacological agent, dyslipidemia, and insufficient knowledge. The most prevalent risk factors of the nursing diagnosis in the studied population were related to insufficient knowledge of modifiable health habits, such as sedentary lifestyle. The information provided is expected to serve as the basis for the planning and implementation of health actions aimed at reducing modifiable risk factors for cardiovascular disease.

## 1. Introduction

Cardiovascular disease (CVD) accounts for 17.7 million deaths per year, which amounts to 31% of all global deaths. Four out of five CVD deaths are due to heart attacks and strokes and more than 75% of these deaths are concentrated in low- and middle-income countries [1]. At the macroeconomic level, CVD implies a heavy burden on the economies of these countries [2], where Brazil, countries on the African continent, and East Timor are some examples. One of the causes for the high prevalence of CVD in these countries is the lack of integrated primary healthcare programs for early detection and treatment of those people with cardiovascular risk factors. This makes access to effective health services that meet the patients’ needs scarcer and, also, unequal. These patients are diagnosed at the end of the disease, and as a result, they die younger, often in their most productive years of life [2]. In the absence of risk factors, epidemiological studies have confirmed that CVD would be a relatively rare cause of death [3]. Therefore, primary healthcare prevention, through research, detection, and management of cardiovascular risk factors, plays a relevant role in the prevention or reduction of these risks, and, furthermore, in the progression and complications of CVD in an effective and less harmfully way [4].

In line with the above, the NANDA-I (North American Nursing Diagnosis Association) taxonomy defines the nursing diagnosis “risk of impaired cardiovascular function” as a vulnerability to internal or external causes that can damage one or more vital organs or the circulatory system. Risk factors for this nursing diagnosis are pharmacological agents, insufficient knowledge of modifiable risk factors, diabetes mellitus, dyslipidemia, sedentary lifestyle, high blood pressure, history of cardiovascular disease, family history of cardiovascular disease, 65 years of age, obesity, and smoking habit [5]. This type of diagnosis arose due to the demands posed by primary healthcare, as nurses care for many chronic patients with various associated diseases, that are under treatment with or dependent on various medications. In these patients, the problems associated with each disease, which are most often cardiovascular events, are not perceived and, therefore, do not receive assistance [6].

These risk factors are widely mentioned in the literature and have long been studied as elements that, isolated, may cause cardiovascular changes. Therefore, they help detect the problem and measure the risk level [6,7,8,9]. This study used the nursing taxonomy of the NANDA 2015–2017, defining a nursing diagnosis as the clinical judgment concerning an individual, family, group, or community response to health conditions/life processes, or a vulnerability for that response. In this way, nurses can diagnose health problems, risk states, and willingness to promote health [5]. Nursing diagnosis is part of the clinical trial of the nursing process [6]. To facilitate some stages, standardized linguistic items are used, such as classification systems that support clinical documentation for professional practice [10].

Early identification of risk factors in exposed individuals will help develop intervention measures that may eliminate and/or reduce these risks and, consequently, prevent the development of CVD. In relation to this, adopting the appropriate prevention measures could reduce the incidence of CVD by up to 80% [11]. Since the main competence of nurses is the identification and control of cardiovascular risk factors, as a rule, the nurse should promote healthy lifestyle habits in the entire healthy or not healthy population, at any age. Therefore, in order to be able to carry out prevention interventions adapted to each person, knowledge, prevention, diagnosis, and control of cardiovascular risk factors is essential.

In this context, due to the increase in some risk factors and cardiovascular events in young adults, it is necessary to investigate the risk of impaired cardiovascular function in Brazilian and international university students. This specific need for research in the young population of Brazil is coupled with another major need. As was identified in a previous study [11], there are currently multiple instruments for identifying and measuring cardiovascular risk, often not in agreement with each other. The possibility of identifying cardiovascular risk with a simple valid and reliable diagnosis covers the need to study the problem from an epidemiological point of view. Furthermore, this represents an important innovation by providing a new nursing diagnosis that is a simple instrument for identifying the problem.

This research aims to verify the accurate use of the nursing diagnosis “risk of impaired cardiovascular function”, by analyzing its clinical validity, specificity, and predictive value. Additionally, we aim to describe the epidemiology of this diagnosis in Brazilian and international university students.

## 2. Materials and Methods

### 2.1. Design

An observational cross-sectional study was conducted. The clinical validity of the nursing diagnosis “risk of impaired cardiovascular function” was addressed by taking the Fehring model [12,13] as a reference, as well as the concordance analysis between observers. This analysis was complemented by the classical epidemiological approach for the assessment of a diagnostic test by calculating sensitivity and specificity, predictive values, and probability reasons.

The Fehring model aims to obtain evidence of the existence of a given diagnosis from the actual clinical setting. The original model used a clinical observation approach, with two expert clinicians doing the observations and the ratings. Clinical experts assess a given number of patients and observe for the presence or absence of each defining characteristic of the diagnosis being validated. Subsequently, the weighted interrater reliability ratios are calculated for each risk factor. Concordance between observers was tested by the Kappa index, therefore, it was possible to analyze the accuracy for diagnosis [14].

Once the clinical validity and accuracy of the diagnosis are proven, this is possible to be reinforced with the sensitivity and specificity analysis of the measure representing the risk diagnosis, as well as with positive and negative predictive values [14,15]. The results underpin the clinical validity of the diagnosis, as seen by the research team.

### 2.2. Setting

**Figure 1:**
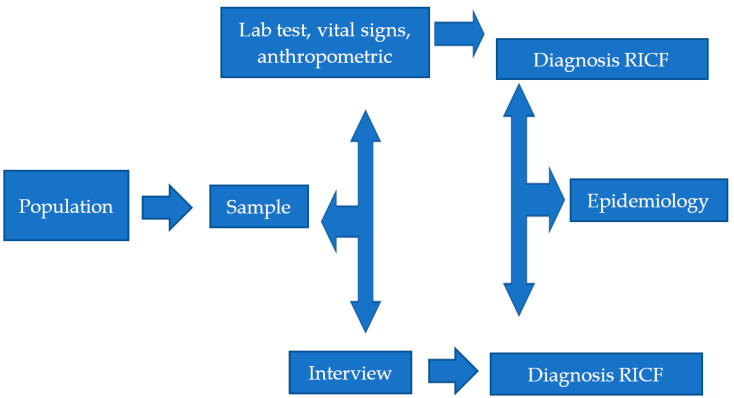
Study methodology flowchart. (RICF: risk of impaired cardiovascular function).

The study site was a federal public university in the state of Ceará, whose campuses are located in the municipalities of Redenção and Acarape. Data collection was carried out through the primary source from January to July 2017. Figure 1 shows the study flow chart.

### 2.3. Sample

The population was composed of 2496 university students of both sexes, over the age of 18, born in the following countries: Brazil, Angola, Cape Verde, Guinea Bissau, Mozambique, São Tomé and Príncipe, and East Timor. They were enrolled in the university and they regularly attended the courses contemplated in the Undergraduate Program. The sample was calculated using the finite sample formula. For calculation purposes, the number of individuals in the population was considered (*N* = 2496), with 95% confidence interval, 50% proportion of the population of individuals with the study problem, so as to maximize the sample size, and with an accuracy of 5%. After the calculation, the required sample was 333 university students.

### 2.4. Methodology

The sample recruitment was carried out through invitation or inclusion of volunteers who agreed to participate in the research. The data collection was conducted through interviews, using a form developed by the research team was disseminated. This form was based on the risk factors of the nursing diagnosis “risk of impaired cardiovascular function” approved by the NANDA-I 2015–2017 taxonomy. Once the instrument was designed, it underwent the previous test with 10 university students and no changes were suggested. It should be noted that these participants were not included in the sample. The nurses who conducted the interview were blind to the objective data.

In addition to the interview, anthropometric data (weight, size, and Body Mass Index (BMI)), blood pressure, as well as laboratory tests results (blood glucose, cholesterol, and triglycerides) were collected. These data were collected as clinical confirmation of the analytical values of cardiovascular risk factors. With the obtained information, the research team designed an independent gold standard combining subjective data obtained in the interview and objective data from the physical examination and laboratory outcomes. The purpose of the gold standard was to verify the risk of impaired cardiovascular function diagnosis risk factors through the cardiovascular clinical parameters, allowing to classify individuals according to the presence or not of the diagnosis.

Considering that the inference of the nursing diagnosis “risk of impaired cardiovascular function” should be made in pairs, ten nurses specializing in the area of cardiovascular health and in the systematization of nursing care, were selected with a minimum of master’s degree and clinical experience in the cardiovascular area or in the nursing taxonomy. Their task was to establish, in pairs, the presence or not of the diagnosis in the study population. Disagreements in classification were referred to a third skilled nurse.

### 2.5. Data Analysis

#### 2.5.1. Clinical Diagnosis Validity: Fehring Clinical Diagnostic Validity Model

Risk factors were defined operationally. Each nurse showed a ratio between accurate and non-accurate diagnoses. Following the Fehring model [12], the reliability ratios may be calculated in the inter-classification.

R = (A/A + D) × ((F1/*N* + F2/*N*)/2)

where R = valued inter-classification ratio reliability, A = number of coincidences, D = number of divergencies, F1 = frequency of the risk factors observed by the first nurse, F2 = frequency of the risk factors observed by the second nurse, and *N* = number of patients observed.

This was done by matching what was valued for each of the risk factors. The formula revision indicates that reliability should range from 0 to 1, where 1 would be the highest possible value. Risk factors with objective laboratory or physical examination data are diabetes, dyslipidemia, arterial hypertension, and obesity. For the rest, the review of cases by the research group was used.

#### 2.5.2. Clinical Diagnosis Validity: Concordance between Observers, Kappa Index

The Kappa index incorporates a correction that excludes matching due exclusively to randomization. To interpret the index value, the following scale is used [16]: k < 0.20: poor concordance, k between 0.21 and 0.40: weak concordance, between 0.41 and 0.60: moderate concordance, between 0.61 and 0.80: good concordance, and between 0.81 and 1: very good concordance.

#### 2.5.3. Clinical Diagnosis Validity: Sensitivity and Specificity Analysis

With the sensitivity and specificity analysis, the measurement representing the risk diagnosis is expanded [14,15]. The measurement validity is calculated on the basis of the information obtained in a double-entry table. The presence or absence of the problem is determined through the positive criterion in individuals with risk, and through negative criterion in those who do not have risk. When interpreting the diagnostic analysis of the risk of impaired cardiovascular function, inferences are made about the existence or not of the problem. This, in turn, can be quantified by using predictive values.

#### 2.5.4. Risk of Impaired Cardiovascular Function Risk Factors

Regarding the interpretation of the results, the World Health Organization recommended considering obesity in case of BMI ≥ 30 [1]. To interpret blood pressure values in the consultation, the tables that were proposed and considered suitable for casual blood pressure measurement were used in people over the age of 18. It was considered hypertension if systolic blood pressure values were ≥140 mmHg and diastolic blood pressure values ≥ 90 mmgHg [17]. To analyze the results of plasma glucose levels, diabetes mellitus was considered if fasting blood glucose was ≥126 mg/dL [18]. The following reference values (mg/dL) for dyslipidemia in adults over 20 years of age were adopted for the lipid assessment: isolated hypercholesterolemia when isolated LDL-c ≥ 160 mg/dL, isolated hypertriglyceridemia when isolated triglycerides ≥ 150 mg/dL, mixed hyperlipidemia when LDL-c ≥ 160 mg/dL and triglycerides ≥ 150 mg/dL, and decreased HDL-c when HDL-c < 40 mg/dL (men) and < 50 mg/dL (women) or associated with increased LDL-c or triglycerides [19].

The Pearson’s chi-squared test was used to analyze the association between the nursing diagnosis, its radiofrequency, and its sociodemographic variables, establishing for all analytical techniques a significance level of 0.05. The collected data were tabulated in an Excel Program for Windows^®^ 2010 (Microsoft, Redmond, WA, USA) for the statistical analysis and processed with the IBM SPSS statistic package for Windows version 22.0, Statistical Package for Social Sciences (IBM, Armonk, NY, USA).

### 2.6. Ethical Aspects

The ethical principles for scientific research were respected. Participants signed the Free, Prior and Informed Consent, and the project was approved by the Research Ethics Committee of the University for International Integration of the Afro-Brazilian Lusophey, with registration number 2.189.523. The study was conducted according to the guidelines of the Declaration of Helsinki.

## 3. Results

To present the results, the sample will be described, as well as the results of the diagnostic validity analysis and the epidemiological results.

As for the sociodemographic profile of the participants, students of Brazilian nationality prevailed: age group of 18 to 24 years (78.4%), women (60.5%), brown-skinned (71.0%), single (90.5%), unemployed student (74.8%), and living with friends (46.8%). Among international students, they were between 18 and 24 years of age (72.2%), men (59.7%), black-skinned (82.6%), single (97.9%), unemployed student (82.0%), and living with friends (86.1%).

As for the characterization of the specialists, the age group was of 26 to 40 years, with an average of 33.3 years. A doctorate in the area of nursing (60.0%) was highlighted with an average of 10.1 years of training and 2 to 6 years of experience (66.6%) in the area of nursing care systematization.

When analyzing the nationality of the participants, Brazilian students prevailed (56.9%), since in the institution where the research was carried out, the greatest number of students were of this nationality. However, it should be noted that 43.1% of the sample was made up of students from other Lusophone countries, covering several countries on the African continent and East Timor.

### 3.1. Clinical Diagnosis Validity

Table 1 describes the values that determine the diagnosis validity. The data source used by expert clinical nurses for the assessment of the presence of a diagnosis is described. Regarding the gold standard used by the research team to assess the relevance of the diagnosis, the data determining whether it was successful is indicated. The key results resulting from applying the Fehring model and the Kappa Index are also included. For the Kappa index, Altman’s data plotting [16] for interpretation of methods was added. Each of these values is disaggregated for diagnostic risk factors, except for the age over 65. In the patient sample, there was no case with that age or higher.

Table 2 summarizes the cardiovascular risk assessment according to nurses’ clinical judgement after interview and physical examination values.

The sensitivity or probability that the measurement correctly ranks the individual at risk was, in this case, 0.99. The positive predictive value was 0.95: this represents the probability that an individual with a positive result may present the problem. That is to say, out of every 100 people diagnosed with risk of impaired cardiovascular function, 95 have the problem. The specificity or probability that the measurement correctly classifies a person as free of risk was, in this case, 1. That is, out of every 100 people excluded of risk, none of them have that risk. The negative predictive value was 21.26, and it represents the probability for a patient with a negative result of not presenting the problem.

Predictive values depend not only on sensitivity and specificity, but also on the prevalence of the problem. In the case at issue, the prevalence was high, so a positive result tends to confirm its presence while, if it was negative, it would not contribute to excluding it. Prevalence is the most decisive factor in predictive values [14,15]. In this study the prevalence obtained was 12.8%.

### 3.2. Risk of Impaired-Cardiovascular Function Diagnostic Risk Factors

Table 3 shows the distribution of university students according to the association between sociodemographic characteristics and the nursing diagnosis “risk of impaired cardiovascular function”.

The risk factors of impaired cardiovascular function in Brazilian students were family history of CVD (93.2%), sedentary lifestyle (56.8%), pharmacological agent (40.0%), dyslipidemia (35.3%), and insufficient knowledge of modifiable risk factors (25.3%). The other risk factors for this nursing diagnosis were low: personal history of cardiovascular disease (12.7%), obesity (9.5%), high blood pressure (4.7%), and diabetes mellitus (0.5%). As for international students, these risk factors were family history of CVD (64.6%), insufficient knowledge of modifiable risk factors (50.7%), sedentary lifestyle (27.1%), and pharmacological agent (20.1%). Other risk factors were poorly present: dyslipidemia (10.4%), personal history of CVD (6.2%), smoking habit (6.2%), hypertension (4.2%), obesity (4%), and diabetes mellitus (0.0%). Table 4 shows the association between sociodemographic characteristics and the nursing diagnosis “risk of impaired cardiovascular function” regarding Brazilian and international nationality.

## 4. Discussion

The data obtained in terms of clinical validity were consistent from the point of view of the Fehring model, Kappa index (although moderate for dyslipidemia), and the analysis of sensitivity, specificity, and predictive values. The data found in this study showed a high level of risk of impaired cardiovascular function among the young population.

These results are consistent with previous studies focused on clinical validation of nursing diagnosis, in which high sensitivity and specificity was obtained. This was the case for some indicators for the identification of nursing diagnosis “ineffective protection” such as fatigue among hemodialysis patients [20], or the indicator “deficient immunity” among adolescents with cancer [21]. Results were also in line with the sensitivity and specificity obtained for the indicators “short stature for age and sex” and “growth velocity less than expected” regarding the nursing diagnosis “delay in growth in adolescents” [22]. However, our results are in contrast with previous clinical validation of nursing diagnosis in which low specificity [23] or low sensitivity [24] was obtained.

The Causation and Validation of Nursing Diagnosis model describes the clinical development of a diagnosis as a temporal process that occurs gradually and where risk factors are considered as the antecedents and starting point for the validation process. Over time, these risk factors consolidate and converge in the essential attributes of the diagnosis, which allow to identify the clinical situation of the diagnosis [25]. According to this model, the present paper identified the most common risk factors in the study population, which could be considered its essential attributes. This model has already been followed for a clinical validation of a nursing diagnosis, identifying the influence of the clinical spectrum to modify the diagnostic inference [26].

In terms of age, the age group of 18 to 24 years prevailed, both in Brazilian university students (78.4%) and in international students (72.2%), with a mean age of 22.7 years. A survey on 4649 undergraduate students from seven Asian countries showed a mean age of 20.5 years, with a standard deviation of 2.9 [27]. In Mexico, the mean age was 20 years, ranging from 16 to 27 years [28]. Considering the prevalence of women among Brazilian participants, other studies conducted in Brazil also presented this characteristic [29,30,31]. The review conducted by Kane et al. concludes that the changes associated with the aging process of the heart and blood vessels differ between the two sexes. Age-dependent cellular, structural, and functional deficits differ between men and women, making them susceptible to different cardiovascular diseases [32]. It is worth highlighting that, in CVD healthcare, the biological peculiarities of each sex should be identified regarding the existing differences in terms of cardiovascular risk factors. Thus, research on this fact should consider the prevalence of these biological peculiarities and how they are presented by different pathophysiological mechanisms among men and women, which affect their perception of health-related quality of life [33]. According to the results, Brazilian students showed a significant higher prevalence of the nursing diagnosis than the international students. This could be explained by cultural differences. The high prevalence of cardiovascular risk among Brazilian population has been previously described [34], therefore, Brazilian students would be more likely to have the nursing diagnosis “risk of impaired cardiovascular function”.

The nursing diagnosis “risk of impaired cardiovascular function” was observed in most university students who had one or more risk factors assessed by the nursing diagnosis. Thus, the importance of investigating this diagnosis in this specific population was highlighted. Results were consistent with a study on Brazilian adults of both sexes, on risk behaviors and related morbidity of diseases in general and non-communicable diseases, two modifiable risk factors prevailed: inadequate diet, and lack of physical activity [34]. It should be emphasized that most CVDs can be prevented through appropriate strategies aimed at addressing behavioral risk factors. More than half of young adults have at least one risk factor for coronary heart disease, which significantly increases the risk of heart disease throughout life. Since several risk factors for cardiovascular diseases arise in adolescence and last into adulthood, prevention should primarily begin in childhood and adolescence [35].

According to the results of this study, the most prevalent cardiovascular functioning risk factor was family history of CVD. These results are in line with those obtained by Vornanen et al., who identified a positive association between the family history of CVD and the perceived risk of CVD, especially among the young population [36]. However, as suggested by the review by Imes et al., the perceived risk of CVD caused by family history is not enough to promote changes in health-related behaviors [37]. Family history of CVD has been associated with increased prevalence of cardiovascular disease and stroke [38]. Moonesinghe et al.’s study identified that the prevalence rate of cardiovascular disease was more than double for the population with a family history of CVD [39]. According to Akhuemonkhan et al., people with a family history of CVD are at increased cardiovascular risk as they are more prone to smoking and obesity [40]. Danilo et al. described the positive relationship between the family history of CVD and cardiovascular risk markers in adolescents such as the Body Mass Index (overweight), high triglycerides, high LDL-C, high total cholesterol, and high fasting glucose [41]. Therefore, as the review by Kashani et al. concludes, family history of CVD contributes to cardiovascular risk and should be incorporated into the cardiovascular disease risk assessment for a more accurate estimate, as was done in this study [42].

The second most prevalent risk factor in the study population was sedentary lifestyle. The degree of sedentarism identified was higher than that obtained in similar studies [43,44]. Evidence showing the relationship between physical activity and cardiovascular disease is broad. In this way, sedentary lifestyle has been associated with worse cardiovascular health indicators such as Body Mass Index, waist circumference, body fat percentage, diet, blood markers (glucose, lipid profile, lipoproteins, and insulin), and blood pressure [44,45]. The review by Del Pozo-Cruz et al. concluded that replacing half an hour of inactivity with moderate-intense exercise reduces cardiovascular risk markers [46]. Lee et al.’s study revealed that people practicing physical exercise had up to 45% lower risk of cardiovascular mortality and higher life expectancy in three years [47]. The recent review by Lacombe et al. described that people with a sedentary lifestyle are half more likely to develop cardiovascular disease or die from a cardiovascular event or disease, with no differences regarding age or sex [48]. According to Whitaker et al., watching television has a stronger negative impact on cardiometabolic risk factors than other sedentary behaviors [49].

The nursing diagnosis “risk of impaired cardiovascular function” encompasses several risk factors that can cause cardiovascular disease, nursing interventions should follow an appropriate approach through various methods such as: telephone counselling, diet counselling, emotional support, hyperglycemia/hypoglycemia control, counselling for smoking cessation, assessing weight loss or weight control, physical activity orientation, health education, and encouraging change in inappropriate behaviors, patient agreement, and active listening [50]. Therefore, the nursing intervention, when performed in young people with CVD risk factors, could prevent, control or delay CVD, as well as complications related to this condition and, consequently, improve the quality of life of this population.

Cardiovascular risk factors are measurable. A recent review compared 23 cardiovascular risk factors measuring instruments, showing the existing variability of assessing tools that facilitate identifying the risk [11]. This, as seen by the research team, endorses one of the central ideas about the diagnosis “risk of impaired cardiovascular function”, its simplicity to describe and identify the phenomenon. The identification of risk factors of higher prevalence in the population enabled to carry out effective cardiovascular prevention programs which resulted in a significant reduction in CVD-associated mortality [51]. The adoption of intervention measures aimed at reducing the prevalence of CVD is directly related to the control of each risk factor, which is generally modifiable. Therefore, the concern and joint effort of health professionals, e.g., nurses, to control and combat the incidence of CVD, which affect the population quality of life, should be imperative [52]. In this sense, according to a recent study [53], greater knowledge is likely to be linked to better practices and more correct attitudes. This is fundamental for the “Millennium generation” and the associated digital readiness, as these contents must be digitalized, made available online for free consulting, and constantly updated [54,55], as is happening with daily information about COVID-19 [56].

As contribution to modifying clinical practice, the results of this study help in the consolidation of the methodological development of the diagnosis “risk of impaired cardiovascular function”. Since its approval and incorporation into the NANDA-I taxonomy, this diagnosis has been used in studies developed in varied clinical settings worldwide, also regarding hypertension and diabetes [57] and hemodynamics [58] in Brazil, and in the field of quality and information systems, such as in the study by D’Agostino et al. [59] in Italy and the one by Pérez-Rivas et al. [60] in Spain. The results of this study reinforce the clinical validity of this diagnosis, especially in the Brazilian population. This is intended to increase the confidence of nurses for their use and to promote their implementation in the detection of cardiovascular risk by this professional group. By assessing the high frequency of the nursing diagnosis, this study identified, by also considering sociodemographic characteristics, the most vulnerable groups and cardiovascular risk factors that manifest in Brazilian and international students’ results. This identification will serve as a basis for the health institution, and especially the nursing community, to plan intervention strategies according to the reality found, based on the Nursing Intervention Classification [61] and the Nursing Outcomes Classifications [62]. In this way, cardiovascular risks may be reduced and the quality of life of the academic community may be improved.

### 4.1. Limitations

As for the study limitations, it is worth mentioning the convenience sample, that limits the external validity of the data. The sample recruitment process was taken as a limitation as it was made through invitation or inclusion of volunteers due to logistical limitations to reach the appropriate number of students per country. In this sense, these data must be checked in other populations of students to confirm an international tendency. It is also noteworthy the inequality of the sample in terms of sex, with more women than men. Although justifiable because a sample of the university population does not have the same profile as that of the general population, this inequality could imply a sex bias to be extrapolated to the general population.

Another limitation related to the profile of the sample has to do with the cultural characteristics of the study population. As it is mostly Brazilian, and Lusophone, nutritional habits, family and personal history, and psychosocial factors related to cardiovascular risk are expected to vary when compared to populations of different characteristics. However, this limitation can also help identify the weight of each of these issues in different populations with respect to cardiovascular risk.

### 4.2. Future Perspective

Clinical validation of the “risk of impaired cardiovascular function” diagnosis consolidates the reliability of the diagnosis. This could motivate nurses to use this resource and further consider it in health assessments, thus allowing them to identify vulnerable people and initiate personalized care plans to make consistent clinical decisions. Further research on cardiovascular risk is needed in young populations, particularly regarding those health-related modifiable factors that are approachable through health education. More specifically, the prevention of smoking habit, alcohol consumption, sedentarism, and obesity should show good results in reducing and controlling the risk. Thus, it is needed to refine the NANDA-I, NIC (Nursing Interventions Classification), and NOC (Nursing Outcomes Classification) taxonomies.

## 5. Conclusions

The nursing diagnosis “risk of impaired cardiovascular function” showed clinical validity and high sensitivity and specificity, as well as predictive values. Results allowed to identify, in a general way and in accordance with sociodemographic characteristics, the presence of this diagnosis among Brazilian and international university students.

The most prevalent risk factors of the nursing diagnosis in the studied population were, among Brazilian students: family history of cardiovascular disease, sedentary lifestyle, pharmacological agent, and dyslipidemia. Regarding international students, the most relevant factors were family history of cardiovascular disease, insufficient knowledge of modifiable risk factors, and sedentary lifestyle. All these factors, independently from the country, are considered of extreme importance by Public Health and Health Education bodies, especially those related with CVD risk. So, educational interventions focused on the prevention of cardiovascular risks are suggested. The availability of online information would be especially useful for its dissemination and updating, given the familiarity of the young population with the use of the Internet.

The information provided is expected to serve as the basis for the planning and implementation of health actions aimed at reducing modifiable risk factors for cardiovascular disease, in accordance with the reality found. Considering that there are few studies on the NANDA-I 2015–2017 “risk of impaired cardiovascular function” nursing diagnosis, these results may encourage other nursing researchers to investigate both the population studied and other groups and consider the interventions and nursing outcomes stated in this diagnosis.

## Figures and Tables

**Table 1:** Risk of impaired cardiovascular function. Clinical validity results and diagnostic concordance.

Risk Factor	Source	GS ^1^	F Ratio ^2^	K Index ^3^	I-K Interpretation
Diabetes Mellitus	Interview/Laboratory value	Laboratory value	1	1	Very good
Dyslipidemia	Interview/Laboratory value	Laboratory value	0.53	0.5	Moderate
Family history of CVD	Interview	Interview	0.98	0.93	Very good
Individual history of CVD	Interview	Interview	0.6	0.72	Good
Arterial hypertension	Interview/Physical examination	Physical examination	0.93	0.83	Very good
Lack of knowledge of modifiable risk factors	Interview	Interview	0.79	0.9	Very Good
Obesity	Interview/Physical examination	Physical examination	0.92	0.97	Very Good
Pharmacological agents	Interview	Interview	0.98	0.93	Very Good
Sedentarism	Interview	Interview	0.96	0.99	Very Good
Smoking habit	Interview	Interview	0.94	1	Very Good
Factors’ mean			0.86	0.96	Very Good
RICF ^4^	Interview/Physical examination/Laboratory value	Interview/Physical examination/Laboratory value	0.89	0.92	Very Good

^1^ Gold standard. ^2^ Fehring ratio. ^3^ Kappa index. CVD: Cardiovascular Disease; ^4^ (RICF): Risk of impaired cardiovascular function.

**Table 2:** Cardiovascular risk assessment.

	Students with Cardiovascular Risk	Students without Cardiovascular Risk
Clinical judgement	*n* = 320	*n* = 14
Physical examination	*n* = 15	*n* = 15

**Table 3:** Sociodemographic characteristics associated with the nursing diagnosis “risk of impaired cardiovascular function” in international university students.

Variable Outcome	Nursing Diagnosis: Risk of Impaired Cardiovascular Function					*p*-Value ^1^
Predictive Variables	Present		Absent		Total	
	*n*	[%]	*n*	[%]		
Age group						
≥25 years old	67	[23.7]	12	[27.3]	79 [23.7]	0.544
18–24 years old	223	[76.9]	32	[72.7]	255 [76.3]	
Nationality						
Brazilian	183	[63.1]	07	[15.9]	190 [56.9]	0.000 *
International	107	[36.9]	37	[84.1]	144 [43.1]	
Sex						
Female	162	[55.9]	11	[25.0]	173 [51.8]	0.000 *
Male	128	[44.1]	33	[75.0]	161 [48.2]	
Skin color						
Yellow, White or Brown	179	[61.7]	11	[25.0]	190 [56.9]	0.000 *
Black	111	[38.3]	33	[75.0]	144 [43.1]	
Marital status						
Married/stable union	17	[5.9]	00	[0.0]	17 [5.1]	0.099
Single	273	[94.1]	44	[100.0]	317 [94.9]	
Living with						
Friends or alone	193	[66.6]	37	[84.1]	230 [68.9]	0.019 *
Family or Partner	97	[33.4]	07	[15.9]	104 [31.1]	
Occupation						
Scholarship, formal, or informal job	67	[23.1]	07	[15.9]	74 [22.2]	0.284
Unemployed Student	223	[76.9]	37	[84.1]	260 [77.8]	

^1^ Pearson’s chi-squared test. * Statistical significance.

**Table 4:** Sociodemographic characteristics associated with the nursing diagnosis “risk of impaired cardiovascular function” in university students, according to nationality.

Nationality	Brazilian (*n* = 190)				*p*-Value ^1^	International (*n* = 144)				*p*-Value ^1^
Diagnosis	Present		Absent			Present		Absent		
	*n*	%	*n*	%		*n*	%	*n*	%	
Age group										
≥25 years old	36	19.7	01	14.3	0.724	31	29.2	11	29.7	0.930
18–24 years old	147	80.3	06	85.7		76	71.0	26	70.3	
Sex										
Female	114	62.3	01	14.3	0.011 *	48	44.9	10	40.3	0.057
Male	69	37.7	06	85.7		59	55.1	27	73.0	
Skin color										
Yellow/White/Brown	159	86.9	06	85.7	0.928	20	18.7	05	13.5	0.473
Black	24	13.1	01	14.3		87	81.3	32	86.5	
Marital status										
Married/stable union	14	7.7	00	0.0	0.447	03	2.8	00	0.0	0.303
Single	169	92.3	07	100		104	97.2	37	100	
Living with										
Fiends or Alone	93	50.8	04	57.1	0.743	100	93.5	33	89.2	0.399
Family or Partner	90	49.2	03	42.9		07	6.5	04	2.8	
Occupation										
Scholarship/worker	47	25.7	01	14.3	0.496	20	18.7	06	16.2	0.736
Unemployed Student	136	74.3	06	85.7		87	81.3	31	83.8	

^1^ Pearson’s chi-squared test. * Statistical significance.

## Data Availability

Data is contained within the article.
